# Social networks, market transactions, and reputation as a central resource. The Mercado del Mar, a fish market in central Mexico

**DOI:** 10.1371/journal.pone.0186063

**Published:** 2017-10-10

**Authors:** Carmen Pedroza-Gutiérrez, Juan M. Hernández

**Affiliations:** 1 Unidad Académica de Estudios Regionales, Coordinación de Humanidades, Universidad Nacional Autónoma de México, Jiquilpan, México; 2 Institute of Tourism and Sustainable Economic Development (TIDES), Department of Quantitative Methods in Economics and Management, Universidad de Las Palmas de Gran Canaria, Las Palmas, Gran Canaria, Spain; Universidad Rey Juan Carlos, SPAIN

## Abstract

Fish consumption in Mexico is considered low (around 12 kg per person per year) and non-homogeneously distributed across the country. One of the reasons for this situation is the scarcity of wholesale selling sites. In this context, the Mercado del Mar (MM), located in Guadalajara city, Jalisco, is the second biggest wholesale fish market in Mexico, with a distribution of about 500 tons per day and a variety of about 350 different species of fish. In this paper, we argue that MM has accumulated social capital, which is formed from two main resources: buyer and seller relationships, and reputation. Specifically, the MM manages a broad and intensive interaction among business actors and the already achieved reputation allows the MM to adapt to market changes. To validate our hypotheses, an empirical study was conducted in 2015 by means of interviews to fish wholesalers in the MM and a sample of their suppliers and buyers. For simplicity we have only considered fresh water fish. We have followed snow-ball sampling as the survey strategy. Results show that the MM has responded to fish market dynamics organizing a complex network of buyers and suppliers whose relationships can be explained in the form of strong and weak ties. At the same time, reputation has been the central resource to build this social capital and also gives place to market transactions. Additionally, the strategic position of Guadalajara city and the well-connected routes have facilitated fish bulking and distribution in the region.

## Introduction

Lack of fish distribution points in Mexico has been pointed out among the reasons why the country is considered as a low fish consumption country. There are about 47 wholesale fish markets located in different central market halls but they are concentrated only in ten different states of the country [1]. One of these wholesale distribution points is the Mercado del Mar (MM) in the city of Guadalajara. This market is the second biggest fish market in the country, after La Central de Pescados y Mariscos La Nueva Viga in Mexico City.

Wholesale markets are main actors for the distribution of food. To talk about wholesale markets is not only to talk about how the transportation, bulking and distribution of food is carried out, but what type of social relations allow to develop these functions, to link social groups and allow local, regional or international exchange. It is necessary to understand how among these groups different forms of social relations regulate everything, and they interact to obtain certain advantages.

Social relations are carried out with the intention to assure market transactions because possessing tangible assets such as preservation and transportation facilities are not the only necessary conditions giving place to fish trading. It is essential to develop the necessary links to be able to use these resources, to have access to seafood and to be able to timely distribute it, to make fish to flow along the value chain.

Recent studies analyze some economic aspects of wholesale fish markets, such as the behavior of buyers and sellers, and the outcomes in the market considering a market organized in a Dutch auction [2]. Considering data from two wholesale fish markets in Italy, Pescara and Giulianova, another study investigates the role of information in the changes of agents’ behavior [3]. The Fulton market in New York, first presented as a highly competitive market where price differentiation leads to imperfect competition [4]; and in a second paper the author presents the case as a centralized market arguing that it exists because of the heterogeneity of fish and the different tastes and needs of customers, and this behavior has led to market imperfections [5].

Probably one of the most detailed studies about wholesale fish markets is presented by Bestor [6] in his work about the Tsukiji fish market in Tokyo, Japan. From a social network perspective, he talks about how asymmetrical flows of information organized the market and how markets are created by the production and circulation of cultural and social capital as well as goods, services and financial assets.

Although contribute to the economic and social knowledge of wholesale fish markets, the studies above do not explain how the nature and structure of social interactions within the network are the source of valuable resources and how reputation is the market base to maintain competitive advantage and the market place itself.

These aspects will be explored in this paper. In particular, we will study how the Mercado del Mar functions in terms of its network of suppliers and buyers, and identify what type of strategies the wholesalers from this market use to maintain their relations with their network partners and keep the market functioning. We use a social network analysis (SNA) to identify the shape of the relations taking place in the market and the resource based view (RBV) of the firm to understand and explain the nature of these social interactions.

We argue that the MM has accumulated social capital, which is formed from two main resources: buyer and seller relationships, and reputation. Specifically, the MM manages a broad and intensive interaction among business actors and the already achieved reputation allows the MM to adapt to market changes.

The role of reputation as driver of cooperative behavior has been largely experimentally and theoretically analyzed in the literature, for example, [7–10] are recent contributions. These studies assume an evolutionary game in a social network structure where every actor can cooperate or defect with their neighbors at every time step. Every individual’s reputation depends on its past cooperative actions and this information is taken into account by the rest of players in the next time step. The results reveal that, in these ideal societies, reputation enhances cooperation. Moreover, under the assumption that links can be broken or created, reputed agents attract more links than not reputed ones [10].

In this paper, we analyze the role of the reputation in the context of buyer-supplier relationships in fish market. In particular, we show how reputation allows building and maintaining the supply chain. The arguments are summarized in three hypotheses, which are tested in the study case of the MM. Through qualitative analysis we look beyond the shape of the network, we identified the nature of social relations and how through social interactions wholesalers are capable to build different levels of reputation. A positive reputation based on trust gives place to create valuable resources such as alliance formation, horizontal collaboration which enhances flexibility and agility while adjust mismatches between supply and demand, and more importantly a positive reputation attracts network partners in the form of strong and weak ties. Thus, we can contribute to the literature of reputation which, in general terms, is not abundant in strategic management or even less in reference to wholesale fish markets, and in fishery science cases about wholesale fish markets are also scarce.

To achieve our objective we structure the paper as follows: We first describe the MM main characteristics, followed by a theoretical framework explaining the nature of reputation used in our argument. The third section explains the methodology used to achieve our objectives, the social network construction and the field work details. The research findings describe the network and present the qualitative information obtained from the market; we finish with a discussion about the different forms of reputation and the lessons learned from this research. In this last section we emphasize the key features of the results and compared them with arguments already presented in literature.

## Study site

### The fish market

**Fig 1:**
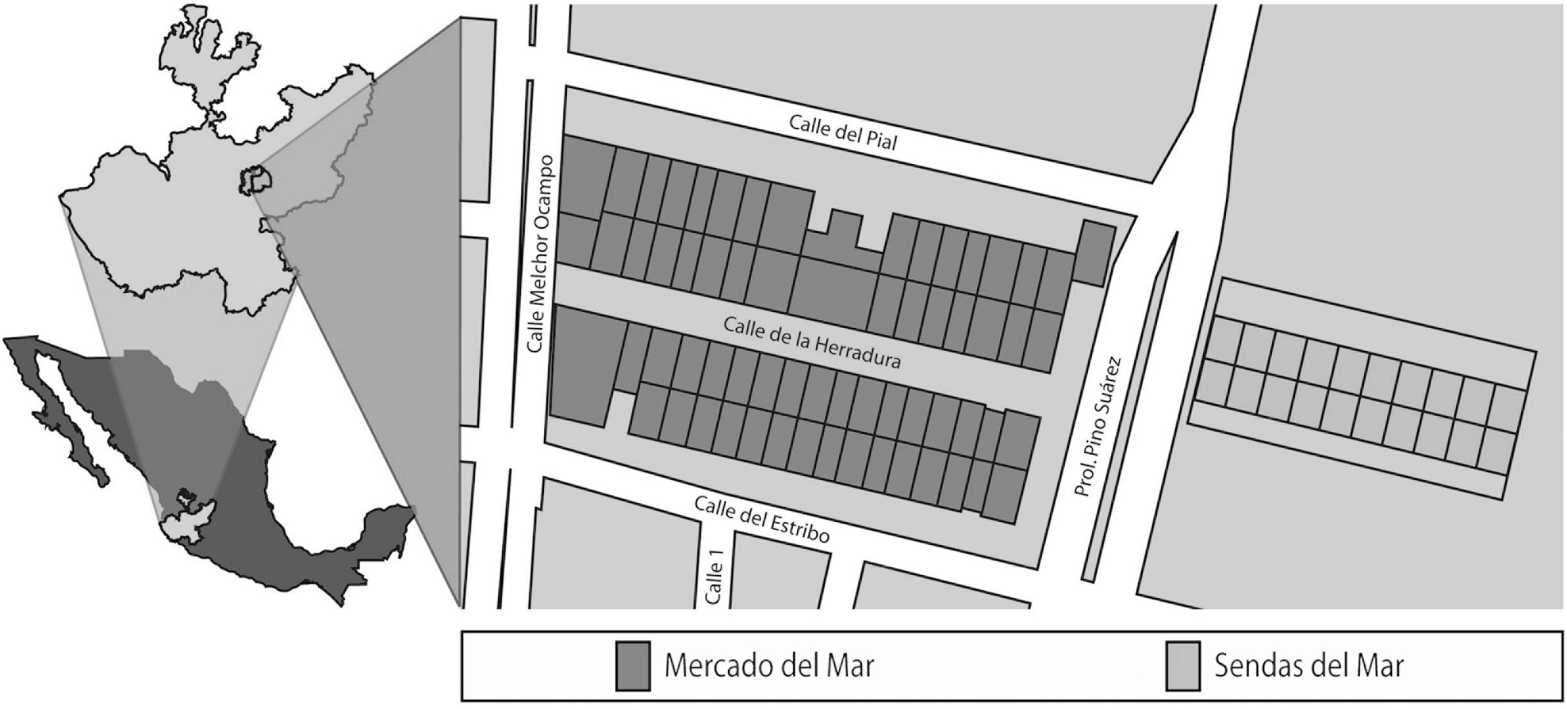
Mercado del Mar, location and structure. Source: Edited by Moises Vargas.

The MM is located in Zapopan, a suburb of the city of Guadalajara, Mexico (Fig 1). Guadalajara is the second largest city in the country with 4 434 878 inhabitants [11]. The creation of the fish market corresponds to the new design of suburban commerce where shopping malls started to develop. In this city this happened during the 1960s and this new model of commerce became common in the 1990s. During this time, Zapopan together with other two municipalities experienced rapid growth at the same time that all Guadalajara’s metropolitan area went through a ‘market growth for corporate grocery stores and construction of many arterial shopping strips’ [12].

The Mercado del Mar is the result of this growth and the trading tradition inherited by Guadalajara’s merchants. Guadalajara’s trading heritage has been developed from the time of its origins to nowadays, ever since the city has played the role of capital and commercial center.

The inherited commercial tradition, the expansion of roads and population growth, gave Guadalajara the necessary elements to specialize as a city to supply services where trading is the largest economic activity that characterizes the city. Nowadays the city is less than four hours drive from the Pacific Ocean and surrounded by lakes and dams, supporting the provision of fresh fish every day. Moreover, the commercial tradition has also created discerning consumers who are used to find a great variety of high quality goods in the different city markets.

These are some of the reasons why the MM was created in 1982 in this city. The MM is exclusively a fish market which operates from Monday to Saturday. The wholesale area opens at four am and closing hours depending on the amount of people buying. It is common that some storage cellars close between nine and 10 am. The retailing section is open from seven to 15 hrs. There are 34 storage cellars, 34 market stalls for retailing, and seven restaurants. In addition to this, there is a linked branch in the same area—called “Sendas del Mar”- that was installed in 2012. This area, in spite of having a different administration, in the mind of customers works as an extension of the MM with 10 more wholesaling storage cellars, eight market stalls for retailing and two restaurants.

In Mexico the MM is number one for the amount and variety of shrimp traded. In fact, it is possible to find there more than 350 fish varieties available throughout the year. There are not formal statistics about its daily sales volume, but most fish traders in the market believe that daily sales to be between five hundred to one thousand tons per day.

## Theoretical perspective

We consider a network perspective of the wholesale fish market based on buyer and seller relationships. As with any organization, a wholesale fish market works under a network structure that has been emerging over a period of time [13]. It is important to clarify that, as stated by Bestor [6], we call market place not only to the market as an economic institution, but also to ‘*a specific geographical place and a localized set of social institutions*, *transactions*, *social actors*, *organizations*, *products*, *trade practices*, *and cultural meaning motivated by a wide variety of factors including but not limited to purely economic or market forces*’ (p. 17).

Additionally, it has also been important for supply management to integrate the network perspective with other management disciplines to facilitate the explanation and understanding of markets’ organization. From the RBV perspective it can be explained how patterns of inter-firm relationships in a supply network can be a source of competitive advantages.

We draw on the RBV theory of the firm to explain why reputation and trust are related and are important resources helping to shape inter-firm networks. The RBV is an approach from the management literature that explains the nature of competitive advantage in firms, how a firm can have different sources of competitive advantage [14]. According to this perspective the base of competitive advantage depends on the resources a firm possesses or control and these can be tangible and intangible. Tangible include financial resources and equipment. Intangible ‘*are deeply rooted in a firm's history and encompass (accumulated) immaterial assets such as patents*, *know-how*, *firm culture or the reputation of a firm*’ [15]. Relational resources are also included in this category and they refer to the inter-firm context, the ties developed with customers, suppliers and other organizations.

We argue that a wholesale fish market is a social network formed by a set of links among a group of individuals. These links present all type of relationships developed by all the actors participating in the market through market transactions, acquaintance, friendship or kinship. These different types of relationships are developed as strong and weak ties [16]. The social relations developed among these individuals are part of the social capital that enables network members to have access to different type of resources.

According to Granovetter [16], ‘*the strength of ties within a network define the strength and quality of relations*’ (p.1361). He explains how the variety of these ties impacts the actions of individuals. Strong ties (ST) and weak ties (WT) can be differentiated through the frequency of interactions. Burt [17] describes weak ties as heterogeneous and critical elements of social structure, enabling information to flow into other social clusters because *‘the value and strength of weak ties is not related to the weakness of the relationship*, *but to the possibility of connections to other social systems*’ (p.63) [18]. In the case of strong ties, they are considered to provide redundant information because they move in similar or the same social circles [17].

### Reputation as a valuable asset

Different industries have different concerns and strategies about how to maintain their place in the market, how to keep the social networks of suppliers and customers that are part of the industry’s functioning, their strong and weak ties. Being perishable, food products in general and fish products in particular require careful handling, and careful marketing strategies must be conducted to continue in the mind of consumers as reliable products. Thus, networks rely not only on the already existing organizational resources but also in resources which can be developed such as trust and reputation.

A major concern in food and fish markets is the health and safety of products. Because food is a vital asset for peoples’ health, consumers need to trust that the product has been well managed and does not present a health risk. In this sense Achrol [19] explains that many business decisions superficially based on trust in reality might be judgments based on a party’s reputation. Therefore, it can be considered that reputation is an element of trust because it affects cognitive perceptions of quality [20], or in other words reputation can be an indicator of reliability [21].

Thus, it is more likely for consumers to trust a company by knowing its reputation. A good reputation will facilitate the establishment of social relations, but if reputation is endangered possible damages can happen to the organization’s expectations. This is why reputation can be considered as a valuable asset [22], because actors with a favorable reputation are more successful in attracting investors, resources, employees, customers and alliance partners [23–24].

Reputation ‘*is a general assessment of the desirable conduct of an organization that is publicly formed and held*’ (p. 54) [25]. Therefore, even though the construction of reputation can be individually built, an organization is in a domain of general interest and has social interactions [24]. These social interactions influence the construct of this concept and the individual building of reputation.

Customers have an image of the firm, depending on its attributes, thus reputation develops on ‘*the enumerated value judgment about these attributes*’ (p. 697) [26]. Reputation is based on credibility and trust and it has to be gained, thus a good reputation has to be well developed and strong [24].

Given that fresh seafood is highly perishable, a positive reputation would be based on the idea of quality equal to the freshness of fish, or as suggested by Graddy [5], the heterogeneity and the different tastes of fish. This and consumer’s needs should be the main dimensions the market has to take care of and present to customers.

Reputation based on quality of the product also depends on consumers’ knowledge about the product, and which elements the consumer considers as quality. In the case of fish, this can most commonly be related to freshness, and less often to variety or product management, such as whether the product is not coming from Illegal Unreported Unregulated (IUU) fishing activities or it has been well managed all along the supply chain. In this case, reputation consists of the clients’ perception of product quality. ‘*Reputation is often derived from the economic notion of perceived quality of current products based on the quality of past products*’ (p. 663) [27]. In other words, if the already known products had good quality it will be more likely that consumers trust the new coming products. Thus, reputation can be seen as a multidimensional construct where elements such as trust, identity, or image can be part of this social construction [28].

Considering this we can suggest a number of research hypotheses concerning the influence of reputation establishment on network formation.

### Reputation establishment

Since we are talking about the market place and the individual institutions that make part of it, we need to have in mind that there is the marketplace’s reputation and each wholesalers’ reputations. However, collective reputations can be transferred to the individuals that are, or have been, members of that specific network [29–30]. Moreover, the elements that serve to construct the reputation of a particular actor may actually be a reflection of the reputation of groups, teams or organizations the focal actor has been involved in [30], because linking to high-quality alliance partners can enhance the reputation of a focal firm especially if the firm is young and entrepreneurial [31].

The MM is compounded by a group of firms, a strategic alliance, where firms are attracted by the market’s reputation to have access to new valuable resources from partners. From the perspective of young and entrepreneurial firms, the affiliation to larger already established alliance partners can provide a range of benefits, such as preferential access to valuable resources or spillover effects from the partner’s reputation [32].

In the case of the wholesalers in the supply chain, it is convenient for them to establish alliances with suppliers with a positive reputation because this means gaining competitive advantage [24]. Moreover, a well reputed firm will positively influence consumers decision making and cause its partners to want to develop a close relationship with it [33]. Thus, the MM’s reputation in general attracts suppliers and buyers, and each firm belonging to this group has to keep its individual reputation in order to be part of the alliance and have positive returns. Considering this we can propose:

### Hypothesis 1

For each company in particular and the MM in general, reputation is established by the number of stable relationships with suppliers and buyers.

Hence, it has been suggested that working with a firm with high reputation can be a competitive advantage because it can improve the image of the purchasing firm [34], therefore we can suggest the existence of different levels of reputation, or different reputations for different aspects, activities or products a firm develops [28]. A firm with high reputation will attract partners, and a firm with a different or a lower level of reputation might attract different buyers or might be less attractive to develop trading transactions with. In other words, the form of the relationship changes according to supplier reputation level. Reputation moderates the relationship between trust and commitment [33].

Thus, it is hypothesized that a positive reputation would be shown in the amount of relationships, strong and weak ties, that shape the network. This means that the larger the alliance portfolios the more opportunities firms can have to a better access of additional resources, knowledge, and information about existing and potential partners, furthermore this would make them more noticeable for firms looking for partnering opportunities [31, 35]. Trust is moderated by reputation [33], thus buyers and sellers who trust the focal firm will be willing to have a relationship with it. We can then suggest:

### Hypothesis 2

For each company in particular and the MM in general, reputation is supported by a large network of relationships with buyers and sellers.

Customer satisfaction has become the main objective in today’s businesses and a key element to maintain a positive reputation. Agility, seen as the ability to respond quickly to sudden changes in supply and demand [36], will contribute to customer satisfaction and consequently to reputation. Horizontal collaboration among network partners could contribute to the agile flow of resources and then to consumers’ satisfaction. Horizontal collaboration occurs when two or more different or competing firms cooperate to share their resources such as ‘*joint distribution centers’* [37].

Horizontal collaboration could be a key factor to enhance agility in the network and the ability to quickly respond to the changing market conditions, diminishing operation risks [38]. Fish trading is a dynamic activity which in order to be able to operate and to have opportune responses to market changes needs to have flexible practices, as well as a capacity to react rapidly to key supply chain outcomes and to develop fast proactive measures. In this way, agility reached through horizontal collaboration provides the capacity to sense opportunities and threats, solve problems, and change the firm’s resource base [38].

In particular, the consumer’s demand for product variety, specifically in the case of short life-cycle products such as food, makes it difficult for manufacturers and retailers to predict which particular variety of products the markets will accept [38]. Demand response needs cooperation with key suppliers to satisfy key customers. Thus, agility enhances trust and customer satisfaction, and as a result is an element to maintain reputation. We can then suggest that:

### Hypothesis 3

The amount of horizontal relationships affects agility and this contributes to customer satisfaction and therefore to the individual and MM’s reputation.

## Methodological approach

### Fieldwork

The fieldwork for this paper was carried out in different visits to the MM, one of the main *tianguis* (open markets) and two fish retailer markets, all located in Guadalajara City. In addition to this there were few visits to three fishing communities who act as suppliers. Visits were carried out from 2013 to 2016.

### Interviews

In this study formal and informal interviews were used. Formal interviews were based on a semi-structured schedule organized around the research questions and theme of study. The form of the emerging data determined the direction and length of the semi-structured interviews. Informal interviews generated additional material and provided a more complete understanding. The objective of the interviews was to find out about the wholesalers links with suppliers and buyers. Our analysis was concerned primarily with common patterns across the supply network structures.

The interviewees agreed to be interviewed, the interviews to be recorded, and they agreed for the quotes that appear in the article to be anonymously published, but they did not agree with the publication of the full interview text in order to safeguard their personal interests. In representation of interviewers in the MM, the market administrator wrote the consent to publish the article in its current form. Ethics and academic quality of this project (IA300215) were evaluated and approved by the Social Sciences and Humanities Coordination at the UNAM.

#### Interviews in the MM

In order to choose a sample in the MM we used a survey strategy based on a non-probabilistic sample adapting chain referral techniques such as response driven sampling (RDS) [39], targeted sampling [40] and snowball [41]. For simplicity the targeted population was the wholesalers selling freshwater fish in the MM.

The first visits were addressed to establish contact with the market administration to explain the aim of our visits and projects, and to find out about the importance, organization and scope of the marketplace. Repeated visits to the MM were made to collect additional data and to note any changes to data collected earlier.

The selection started based on the information obtained from the manager, who was able to indicate the wholesalers trading fresh water fish. Each person interviewed would indicate us those commercializing fresh water fish. Each interviewee indicated other eligible participants from their own social network, and as snowball technique does not limit the number of participants [41], the limit of merchants to be interviewed would be set by those who trade freshwater fish. In this way we tried to cover all segments of the targeted population and to produce a representative sample that would provide useful data on the nature of network ties. The interviews were addressed to the owner or person in charge of the business. Each firm was visited between two and three times to be interviewed for the first time and then to corroborate information. From the 44 wholesalers located in the marketplace, we took a sample of 10.

Chain referral techniques are commonly used for hidden populations. Although this is not a hidden population, we made use of ethnographic techniques to gain access to the merchants’ population, because of concerns over maintaining commercial confidentiality.

#### Interviews to suppliers

Since it was not possible to visit all suppliers, most data were obtained from the interviews with wholesalers and verified in some cases with suppliers. Thus, we were able to triangulate part of the information by visiting eleven processing plants in three fishing communities riparian to Lake Chapala, where most local tilapia filets come from. The intention was to collect data on the upstream and downstream network.

#### Interviews to buyers

*Tianguis (open market) and retailer markets*. In the *tianguis* we interviewed eight market stalls and in the retailer market three market stalls (Table 1). We decided to interview buyers in these places because they are the most loyal and frequent midlevel wholesalers. The aim was to triangulate the information already obtained from the MM wholesalers.

**Table 1:** Interviews to wholesalers and some retailers.

Place	Number of interviews
MM	10 wholesalers 4 administrative personnel
Tianguis	8 market stalls
1 Retailer markets	3 market stalls
3 Fishing communities	11 processing plants
Total	36

### Social network measures

This paper makes use of the social network analysis (SNA) to obtain measures of relationships in the MM. According to this approach, a network is a set of vertices (nodes), edges (links) and the way both elements are connected. In the context of the MM, nodes are represented by the agents (suppliers, wholesalers and retailers), while edges represent trade relationships among those agents. SNA provides some node-level and network-level metrics of the structure and performance of social networks and has been extensively applied to analyze relational characteristics in Social Sciences [42, 43].

**Fig 2:**
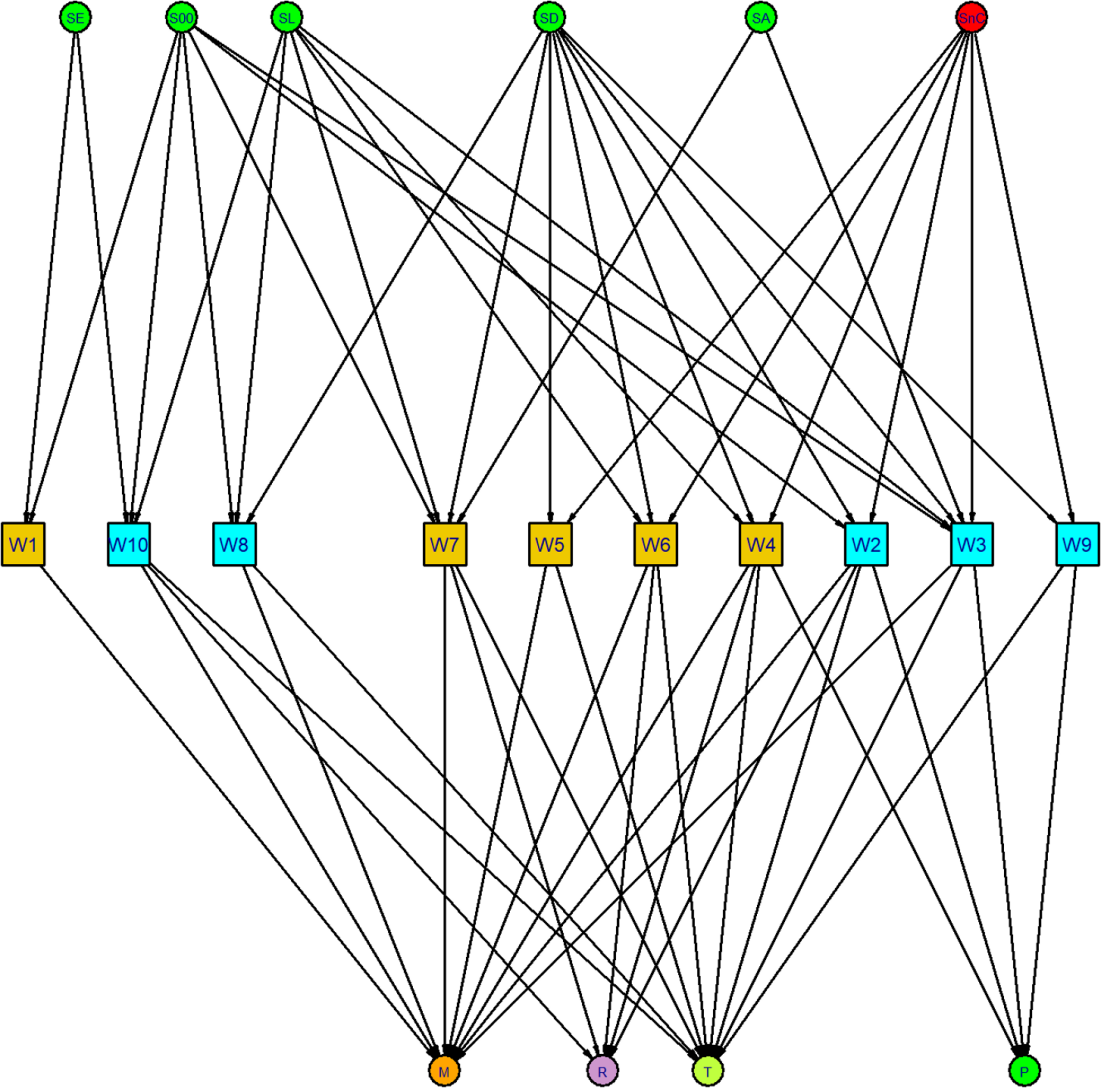
Seafood supply network throughout ten wholesalers in Mercado del Mar, Guadalajara, Mexico. The wholesalers (10) are the square box in the middle and with two colors. The gold/cyan color indicates that the wholesaler does/does not have horizontal links with other wholesalers The circles represent suppliers and buyers, aggregated by sectors. The suppliers are colored according to this classification: classified (green), which are S00-general suppliers, SE-foreign suppliers, SD-dams, SL-lakes, SA-aquaculture, and non-classified (red). The retailers are classified in: M-markets, T-*tianguis*, P-fish shops, R-restaurants, and are colored accordingly. Details can be found in Table 2. Both strong and weak ties are represented with solid lines.

Formally, a network *G = (N*, *L)* is a group of nodes *N = {1*,*2*, *… n}* and edges *L*, which are elements in the domain *NxN*. A network can be graphically represented indicating the nodes with dots (or any geometrical figure) and the edges with segments connecting the dots (see Fig 2). The network can be weighted (if there is some weight in the edges) and directed (edges between nodes have a determined direction represented by an arrow). In the case of seafood trade studied in this paper, an edge between two firms indicates the existence of some flow of fish from a seller to a buyer firm. However, the quantity of fish is unknown. Instead, the edge includes a dichotomic category, which informs about the tie strength between firms. An edge is a strong tie (ST) if the seller provides periodically a quantity of product to the buyer and a weak tie (WT) if trade relationships between parts are casual or sporadic. Therefore, the network in the case study is directed and unweighted, but includes two types of edges, ST and WT.

The algebraic representation of the network is made with the adjacency matrix *A*_*nxn*._ The element *a*_*ij*_ of this matrix, with (*i*,*j*) ∈ *NxN*, adopts value 1 if there is a ST from node *i* to node *j*, -1 if there is a WT between these nodes and 0 if there is not such an edge. Based on the adjacency matrix, it is possible to calculate a series of measures which inform about the topological properties of every node and the whole network. The network metrics used in this paper are described below.

One of them is the node degree, which informs on the number of connections from and to every node. In a directed network, a node *i* has two types of degrees: a) Node *i*’s in-degree ($k_{i}^{in}=\sum_{k=1}^{n}\left|a_{ki}\right|$), which is the number of edges directed to node *i*, and b) Node *i*’s out-degree ($k_{i}^{out}=\sum_{j=1}^{n}\left|a_{ij}\right|$), which if the number of edges from node *i*. In the seafood trade context, node *i*’s in-degree indicates the number of suppliers of firm *i*, while the out-degree indicates its number of buyers, independently of the tie strength. Node *i*’s degree is the sum of its in and out-degree ($k_{i}=k_{i}^{in}+k_{i}^{out}$). These metrics will be used to check H2 in the case of the MM.

A network-level metric is the (in or out) degree distribution (*p*(*k*)) and indicates the proportion of nodes with *k* degree. According to the functional form, the degree distributions can be classified in two types: a) Homogeneous distributions, such as Poisson or exponential, where the nodes in the network have similar degrees around a mean value, and b) Heterogeneous distributions, such as power-law, where a small but significant number of nodes with high degree are combined with many of them with small degree. The latter distribution represents many real networks from Physics, Biology or Social Sciences and its properties have been extensively studied [44].

Starting from the nodes’ in and out-degree, a centralization metric is calculated. It is based on the one proposed by [45], which adopts the following expression:

$$C_{D}=\frac{\sum_{i=1}^{N}\left(k^{*}-k_{i}\right)}{{max}_{k^{*}.k_{i}}\sum_{1}^{N}\left(k^{*}-k_{i}\right)}.$$

The parameter k^*^ is the maximum degree of nodes. Centralization *C*_*D*_ varies from value 0 when all nodes have the same degree to 1 when the sum of differences between k^*^ − *k*_*i*_ is maximum. In undirected networks, this is the case of a star network, where every node has only one link and a single node is connected with the rest. The intermediate values inform about how the relationships are concentrated in few nodes. In the context of supply chains, this centralization metric has been interpreted as “*The extent to which particular focal firms control and manage the movement of materials in a supply network*” [46]. Therefore, it may be used as an indicator of differences in reputation levels among firms in the line of H2. In our study, we analyze the centralization level of the wholesalers in their relationships with suppliers ($C_{D}^{in}$) and retailers ($C_{D}^{out}$).

The last node-level metric used in the case study is the percentage of ST over the total relationships of a wholesaler. This metric informs on the proportion of stable relationships of a wholesaler and will be used to check H1 in the MM. In mathematical terms, the percentage of ST for a node *i* (%*ST*_*i*_) is calculated using the following formula:

$${\%ST}_{i}=\frac{number\;of\;ST\;of\;i}{k_{i}}.$$

Accordingly, it can be also defined the percentage of ST over the total relationships with sellers (${\%ST}_{i}^{in}$) and with buyers (${\%ST}_{i}^{out}$) by restricting to the edges coming in or going out from node *i*, respectively.

## Results

### How does the MM’s social network function?

In this section, we present the wholesale fish market network: Buyers and sellers. This market works under a network structure which has been emerging over a period of about 34 years. In Fig 2 we present the sample of agents aggregated by sectors and the network graph of actors, also aggregated by sectors, who form part of this network. Fig 2 shows those sectors which wholesalers declare to trade with. Thus, the fish market network is a directed bipartite graph formed by wholesalers on the one hand and suppliers/retailers on the other hand. The number and type of links with suppliers and buyers, as declared by wholesalers, are included in Table 2, which contains the statistical results from the network analysis.

**Table 2:** Statistical results of the seafood supply network in the MM. The results are calculated from the total strong and weak ties with firms declared by the wholesaler. The first two columns of numbers indicate the percentage of strong ties (stable relationships) over the total ties of every wholesaler and MM (%ST). The following two columns indicate the total number of relationships with suppliers and retailers of every wholesaler and MM ($k_{i}^{in}$ and $k_{i}^{out}$). The last column gives value 1 if the wholesaler declares the existence of transactions with other wholesalers (H3).

Wholesaler		H1		H2		H3
	Code	%ST with suppliers (${\%ST}_{i}^{in}$)	%ST with retailers (${\%ST}_{i}^{out}$)	#links with suppliers ($k_{i}^{in}$)	# links with retailers ($k_{i}^{out}$)	Horizontal links (HL)
	W1	90,0%	96,1%	20	1145	1
	W2	76,9%	57,1%	13	35	0
	W3	90,9%	23,1%	11	195	0
	W4	66,7%	30,0%	6	50	1
	W5	100,0%	12,3%	6	57	1
	W6	20,0%	36,4%	10	55	1
	W7	66,7%	30,0%	9	50	1
	W8	71,4%	0,0%	7	75	0
	W9	100,0%	-^2^	3	0	0
	W10	75,0%	100,0%	4	10	0
	Mean	75,8%	42.8%	8,9	167	50%
	standard deviation	23,3%	35.1%	5	348	
	Min	20,0%	0,0%	3	0	
	Max	100,0%	100,0%	20	1145	
	Centralization with suppliers ($C_{D}^{in}$)^3^	0.16				
	Centralization with retailers ($C_{D}^{out}$)^3^	0.65				
	Corr. Coef. Supp-Ret	0,07		0,82		
	Corr coef. %ST-#links	-0,04	0,51			
	Mean (HL = 1)	68,7%	41,0%	10,2	271,4	
	Mean (HL = 0)	82,8%	56,0%	7,6	63	
MM^1^	MERCADO DEL MAR	75,3%	73,7%			50%

^1^ The estimations for MM are calculated from the sample of ten wholesalers.

^2^ Since the number of links with retailers is null, this value cannot be calculated. It was omitted for the calculations of correlation coefficients.

^3^ In the calculation of the centralization index, we assume that sellers and buyers are exclusive for every wholesaler. In other words, every firm supplies to only one wholesaler and every retailer buys to only one wholesaler.

### Statistics and working model

The MM as a whole presents good and similar results in terms of percentage of ST with suppliers and retailers (above 70%). This may be an indication of good reputation levels in terms of H1.

The distribution of %ST with suppliers is quite homogenous among wholesalers. There is only one wholesaler (W6) who declares few number of ST with respect to total ties. This may be an indication that reputation levels of every wholesaler in the MM in terms H1 are rather uniform and high.

We give an explanation of these statistical results in terms of what we found in the interviews. According to them this market has grown and strengthen its capacity, first by attracting more wholesalers interested in participating in the already existing network structure, as stated by the market administrator: “*some wholesalers were former suppliers for the MM but they saw an opportunity to establish their businesses here*, *because here you only have to put out the product and is sold*” (Market administrator, 2015). And second, the benefit of gathering this amount and type of wholesalers is that they specialize in different types of products and presentations. This results in the fact that “*in the MM you can find a great variety of fish*, *about 350 species along the year”* and *“people know that they can find fresh fish in the MM*, *fresher than in other states*” (Market administrator, 2016). We can then suggest that the already known reputation of the MM attracted wholesalers to establish their fishing business in the market place, and variety and freshness of fish are the main motives for buyers to participate in this network.

This was verified with the interviewed *tianguistas (open market people)* who responded that the reasons why they always go to buy to the MM are quality, variety, freshness and price.

Wholesalers explained that every early morning the *tianguistas* go around the market place looking for the best buy of the day. “*The tianguistas are those who mostly maintain the market*” (W7). Thus, *tianguis* are part of the ST, the stable relations, with the market place.

“*Clients always return*” (W1) this means that there is a level of trust with buyers which encourage them to continue buying in the MM, this trust generates value in the sense that at some point a client will return to continue trading.

Thus, the opportunity to belong to an already established network has been an opportunity for wholesalers to become business partners in this market. They have benefited from the already stablished reputation from the MM (H1). Some of these benefits consist in the fact that retailers and final consumers already know that the MM is one of the best places where you can find fresh fish, thus newer wholesalers have already gain a positive reputation just because of the fact of establishing in the market place. At the same time, they have built an important everyday bulking and selling capacity. In the interviews they said that the MM sells between 500 and a thousand tons per day, however there are not official statistical records to prove this statement.

Another example that a positive already established reputation will attract suppliers and buyers is the additional branch of wholesalers and retailers established in the market newer addition ‘Sendas del Mar’ (Fig 1). They decided to install their fishing business in this place because people already know that in this area you can find good fish, and buyers not even make a difference between this area and the larger Mercado del Mar. All the market place is a Mercado del Mar for suppliers and buyers, and all of them benefit from sharing a group reputation. Moreover, in Guadalajara every fish market is called “mercado del mar” by consumers, even though the only one that is officially named like this is the one we are referring to. One of the reasons is because the Mercado del Mar was established before the other markets and the MM also partially supply them. This means that even other fish markets, in other parts of the city, benefit from the MM reputation.

The distribution of the number of links across the different wholesalers is more heterogeneous. It presents quite a large variance, with few wholesalers with many links (W1, W3) and the rest with much smaller number of links. This fact is more pronounced in their relationships with retailers than with suppliers, such as it is observed when comparing the centralization index with suppliers (0.16) and with retailers (0.65). This may be an indication of different reputation levels in the MM, especially in their relationships with retailers.

What W1 has in common with his network partners is that the base of his reputation is product quality, to present a product which fulfills clients’ expectations. They are specialized in frozen tilapia and pangasio. However, they have assured that they have the best quality frozen fish in the market, and at the same time they have variety. This statement is based on the fact that the fish they sell is frozen with a lower percentage of water and they sell it for a better price than the one offered by wholesalers in other markets. In addition to this, tilapia is the most consumed fish in Mexico. This also explains the large number of buyers.

W3 assures that his clients look for his products because he has very good quality products, meaning fresh and good variety of fish. W3 explained that “*I have the reputation to have the best fish in the market*, *I receive the best fish*, *and therefore my clients do not mind the price*”.

We triangulated this information with retailers, *tianguistas*, two of them mentioned that they always buy from W3, the rest go every day to buy the freshest and best fish in the market. In other words, this means that one of the reasons why W1 and W3 have a larger number of links is because they have a higher reputation level with retailers. Many *Tianguistas* and most retailers in their daily rounds end up buying with these wholesalers, instead of W10 for instance who also sells frozen tilapia and reports a lower number of links.

In the case of W9 he assures that ‘*I do not have fix clients*, *no one has fix clients*, *all of them come and buy wherever they like the fish each time*’. This statement does not match with the other wholesalers’ opinions; thus we believe the reason is a different reputation level. The fact of not having ST with clients might mean having a lower reputation level or that he has not been able to establish a positive reputation, or any type of reputation among retailers. This wholesaler did not show any particular characteristic which could distinguish him from other wholesalers, or give him elements for strategic advantage. He only sells local freshwater fish like W4 and W7 who have a larger amount of suppliers and buyers. In addition, he does not have horizontal relationships, what might mean a lack of managerial abilities to implement reactive and dynamic strategies to satisfy his clients, a lack of agility.

Half of the wholesalers had horizontal relationships. This fact is a sign of good collaboration levels and shows the way wholesalers have access and benefits from network resources. Each business specializes in particular types of products, thus, when a regular customer comes to buy and the wholesaler has every product but one, he makes use of his market strategic alliances and ask for this particular product to another wholesaler increasing responsiveness in product availability.

Another form of horizontal collaboration is when wholesalers have an unexpected increase in demand and they do not have enough products to supply it. Then they also make use of their links with other wholesalers and borrow products. They will pay back either in cash or returning the same amount and type of fish, or the type of fish the lender wholesaler will ask at the moment of repayment. These are strategic reactive actions giving place to horizontal collaboration to respond quickly to customers demand to always fully satisfied them (H3).

Descriptive statistics in Table 2 provide additional information:

a) There are more links with retailers than with suppliers. This observation can be explained because some of the producers are associations such as fishers’ cooperatives. Thus one supplier is actually representing an organized group of people, meaning that the reported supplier is not a single person but rather a sub-network of producers supplying fish and represented by a network member in charge of bulking fish.
b) Those wholesalers with many links with suppliers tend to have many relationships with retailers. This statement is given by the correlation coefficients, which informs that there is quite strong relationship between the number of links with suppliers and retailers (0.82). This is an indicator of wholesalers with good upstream and downstream reputation, with both suppliers and buyers. One common issue that wholesalers mention during the interviews was that a way to maintain a constant and relievable relationship with suppliers is by assuring a transaction. In other words, suppliers will assure a reliable supply if the purchasing firm commits to always buy their products and to pay them fully. Unpaid debts are a common problem in this business environment, therefore, it might also be an indicator of reputation, and an enhancer of trust and reliability. The same criteria is applied from wholesalers to retailers, they mention ‘*we prefer to sell to Tianguistas because they pay in cash*, *but not to restaurants because they are not very good for covering their debts*. *A similar situation happens with supermarkets*, *they take too long to pay*, *this is why we do not want to do business with them’* (W1, W2). *Tianguistas* also have a high reputation level as the most frequent and reliable buyers in the market, because in addition to frequency they do not create debts.
c) ST are more generalized with suppliers than with retailers. Several reasons can be given for this observation. One of them is kinship, three of the wholesalers are partially supplied by their relatives, either because they are in charge of bulking the product from the cooperatives, or because their relatives have fish processing plants (W4). One of them mentioned that he has his own processing plants in China (W1); and the others have developed a long-term relationship with their suppliers because they maintain good prices, quality and quantity. Among the important quality features they mentioned was taste and size, ‘*we buy from them because their fish has good taste and size’* (W7, W8, W9, W10).

ST with retailers are less common, but they are developed under the bases of trust. Wholesalers mentioned that the buyers they have developed ST with is because their payment is reliable and they are frequent buyers. To keep relationships with these good buyers, wholesalers give them better prices and put aside the best fish for them. Thus, priorities in resource allocation and special prices have helped wholesalers to develop strong ties with frequent buyers. One of the wholesalers explained “*we call our regular customers when we have the product they like*, *hence we put aside the best products for them and give a preferential price*” (W2, 2015, 2016).

d) Those wholesalers who declare to have horizontal relationships have lower percentage of ST than those who say not to have any horizontal relationships. On the contrary, those wholesalers who declare to have horizontal relationships have a higher number of links with suppliers and retailers than the latter.

The latter observation can be interpreted in the sense that horizontal relationships (H3) enhance the number or links (H2). This observation involves two different ways to manage reputation. In the case when horizontal relations imply higher number of links these wholesalers would have a more agile system to satisfy clients’ demands. They use multiple sourcing strategies to achieve an agile flow of fish products increasing customer satisfaction.

At the same time, a lower percentage of ST means that the few ST wholesalers have are reliable suppliers and buyers who cannot be left out of the social network because they assure a reliable supply and, in the case of suppliers they are either their relatives or have developed long-term relationships as already mentioned. In the case of ST with retailers, frequency and reliability in transactions have developed stable relations. Moreover, horizontal relations act as a backup mechanism to diminish risk in case of an unexpected change in demand. Therefore, they are used when needed, acting as latent knowledge and resources when they are inactive.

These last two statistics are also showing the strength of weak ties and the redundancy of ST. Weak ties mean a higher possibility of connections, a larger number of links, more suppliers and buyers, that even if they are not frequent they will always go back. This type of collaboration, horizontal relations, is producing a good customer service, customer satisfaction and a positive reputation shown in a larger amount of links with suppliers and retailers.

The nature of weak ties with suppliers can also be explained when wholesalers buy products on consignment according to demand, mostly from middlemen who act as sporadic suppliers. In this way the risk of losses are only for the supplier and not for the wholesaler, because the wholesaler only pays for the fish already sold. This gives an opportunity to wholesalers to avoid the risk of overcapacity, to have a wider variety of fish and a higher volume in case of unexpected demand, allowing them to better adjust mismatches between supply and demand. This practice is most commonly observed during Lent when fish demand increases due to the Catholic tradition of eating fish on Fridays during this period.

On the contrary, those wholesalers who have no horizontal relations need to have a larger number of ST because they do not have this backup mechanism to face unexpected changes in supply and demand. Thus, they only rely on their ST and lack horizontal cooperation strategies, which also mean a lack of agility to satisfy customers demand for product variety leaving out some of the infrequent suppliers and buyers, and reducing agility to satisfy clients, shown in the lower number of links.

Finally, the MM has a large network of suppliers because its wholesalers look for variety and quality of fish, suppliers come from at least 8 different states in the country including fish coming from the sea, dams, lakes and aquaculture, in addition to the imported fish coming from China and Vietnam.

The large amount of buyers is explained by a wholesaler’s statement “*we sell to all who sell*, *this market supplies all other markets in the city and in many other cities*” (W10, 2015, 2016). They have been able to establish a long and varied network of distribution channels in the city and in other states. In the interviews wholesalers declare to have buyers from 14 different states in the country, in addition to the *tianguis*, local markets and restaurants (H2). Moreover, Guadalajara’s strategic position and trading heritage facilitates the flow of products in and out the region.

## Discussion

The reputation of the market place built over the 34 years of existence has attracted new fishing businesses. This affiliation has provided to newcomers valuable resources or spillover effects [32]. Some of these mutual benefits have been delivery reliability, because both sporadic and constant suppliers would find it easier to go to a market place where they can deliver their goods to one or several partners at the same time, in the same area, instead of delivering in different places increasing delivery costs. Responsiveness to targeted markets, they respond to their client needs by presenting an important variety of fresh products; and they have widespread distribution coverage because buyers know the large variety of products they can find in this market.

Establishing a business in this place means to instantly acquire or benefit from a positive reputation, gaining a competitive advantage [24], because consumers’ social construction of the market is as a place where you can always find fresh fish to buy. Newcomers will benefit from this by having access to the network of suppliers and customers which have been attracted by the positive reputation of the market place.

However, in order to preserve the reputation of the market place more recent newcomers have to keep the necessary qualities to enhance group and individual reputation, because suppliers and buyers would find difficult to discern the individual contributions to the collective performance [32, 34]. A negative individual reputation might affect the market’s reputation as a whole, not only an individual partner [33]. Furthermore, a newcomer has to follow the basis of the collective reputation in order to remain as a network partner. Therefore, we can suggest that new partners by becoming part of the market place acquire reputation by affiliation or association [32].

The ability to form alliances and sub-alliances by network members also demonstrates the alliance creation experience and the capacity of wholesalers to benefit from their experience, since they have been able to link with the right partners and to acquire the right products at the right time. In this case, experience has created value from firm’s alliances [47]. Thus, the acquired reputation, experience and alliance formation have been key elements to attract suppliers and buyers and to develop a network of weak and strong ties.

Some regular buyers such as the *tianguistas*, benefit and use the fact that they are MM clients to build their own reputation. To be able to sell to final consumers, they need to have a positive reputation, which in part is constructed by assuring that fish is daily bought in the MM, proving that working with a high reputed supplier improves the image of the purchasing partner [34].

The constant quality of products, fish freshness and variety, creates trust in buyers, and the ability of wholesalers to bulk and sell a large volume of fish also demonstrates that suppliers trust wholesalers generating a valuable commitment between both parties [48], increasing frequency in exchange which can be seen as a way to develop strong ties.

Strong ties with buyers are built upon source loyalty, when a very small number of people in the buying and supplying firms are actually involved in a transaction. These individuals interact and build up confidential, complex and enduring relations, explaining the nature of strong ties [33]. At the same time, source loyalty is enhanced by high asset specificity, because some wholesalers select and put aside the best products for their most frequent and loyal customers. This demonstrates asset specificity because a partner with a high reputation level enhances trust through the willingness to adapt and invest in the relationship and adaptation of the clients’ requirements.

For the individual wholesalers weak ties in the form of retailers, particularly *tianguis*, represent the value and strength of weak ties not because the weakness of the relationship, but as stated by [18], because the possibility of connections. Most of them buy every day where they find the best catch and pay in cash, having weak relations with individual wholesalers but strong ties with the market place. The *tianguis* are the most frequent clients in the MM. This type of weak ties for individual wholesalers motivates competition and influences each individual wholesaler to maintain quality, variety and prices.

The argument presented in the literature is that the effectiveness of a network depends on both strong and weak ties since different forms of ties provide distinct and different resources [49]. Even inactive ties remain within the network as latent knowledge and resources [16]. In the MM even sporadic customers go back to buy fish, they might be infrequent but they always return to this market place.

The variation in the number of links was explained as different reputation levels because customers will be more loyal, or will look for transactions with those wholesalers with a high reputation, those who have the freshest fish. Different reputations have been expressed in different sub-networks of suppliers and buyers where reputation moderates different levels of trust and commitment [33]. A wholesaler with a higher reputation level will have broader alliance portfolios giving access to more network resources [35], this will help to better satisfy his/her customers, make him/her more attractive for buyers and suppliers to be willing to develop relationships with.

In addition, one of the most important strategies that enhance and sustain the MM’s reputation is horizontal collaboration. This is part of the strategic alliances carried out in this network, and a way to have access to valuable network resources from different partners when needed. A percentage of 50% of the interviewed wholesalers declared to have exchange with other wholesalers in the market place whenever they need to cover a particular customer order or when there is an unexpected change in demand. The frequency of exchange is determined by supply and demand changes. Therefore, horizontal collaboration is giving flexibility to firm’s resource stock and enables them to earn relational rents [49]. Furthermore, this collaborative strategy is enhanced through flexibility and agility which develops responsiveness improving customer services [38] and therefore the market place reputation.

This type of relational capital has been shaped by clients’ demands and the fact that fish is a perishable good. It is a reactive behavior that has helped to build an alliance portfolio among network members, a sub-alliance inside the market place which speeds fish flow and customer service enhancing individual and general competitiveness [31].

For wholesalers horizontal collaboration has also been a strategy to adjust mismatches between supply and demand, and avoids costs which might even be higher in perishable goods such as fish. When is necessary to satisfy demand or supply changes wholesalers collaboration has been proved to be more effective than working in isolation [37]. No doubt that this joint work is an opportunity to improve customer services and probably to lower operating cost [50]. It is difficult that one wholesaler will have on its own all the variety of products that the market offers, however, through horizontal collaboration network partners can obtain and offer the required products when needed. This will increase responsiveness and flexibility resulting in consumers’ satisfaction, as well as individual and alliance gain of value.

To be effective in matching demand with supply, horizontal relations need to take place and network partners need to collaborate. General cooperation observed in the sharing of resources among individual partners in the market has led to operators responding quickly to sudden market changes, to the agile flow of resources and to satisfy demand [36].

Finally, some of our findings in the case study agree with previous experimental results in evolutionary games. The second hypothesis, which states that reputed agents attract new relationships, have been also observed in the experiment conducted in [50]. Additionally, the case study analyses the role of other factors, such as weak/strong ties and horizontal relationships, which are not included in the previous experimental games.

## Lessons learned

The MM is a market place where different types of relationships have developed a social network in which every actor benefits from participating in this alliance, because social relations allow all members to have access to different type of resources, giving the bases to keep the market functioning.

The individual businesses have managed to establish their links with suppliers based on four main factors: ownership of resources, buying, distributing and paying capacity. In addition to a trading tradition and years of experience in the fishing business. Thus, they have strong ties with suppliers because some of them have their own processing plants, or vessels to go fishing. Furthermore, some wholesalers have exclusivity with some suppliers because the wholesaler will assure to buy all their production for a good price.

According to what we learned from the MM way of functioning we can say that the MM reputation is based on and functions because of:

1) Wholesalers experience and ability to have a constant flow of fresh products. This is probably one of the most valuable resources, which enhances individual and market reputation, attracting an important amount of retailers.
2) Wholesalers have developed commitment with the market itself. They are permanently placed there which means that in order to keep their network of suppliers and buyers they have to maintain a strong tie with the market and their individual and market reputation.

We observed different individual reputation levels which are also a necessary part of the market place. At the same time, different reputation levels might enhance competition and a willingness to improve each individual business.

3) Guadalajara’s strategic geographical position and trading heritage is also an asset for the market’s place competitive advantage. Market history and reputation has been built through a long trading experience based on wholesalers heterogeneity, agility to satisfy clients’ needs and the flow and variety of products that pass through the market place. These elements, history and experience, influence directly and indirectly in clients’ behavior and determine the amount of buyers and sellers willing to make transactions in the market place.
4) The existence of horizontal collaboration among wholesalers. This has proven to be a resource to satisfy clients’ demands, to adapt to mismatches between supply and demand, a base to form alliances and a way to adapt, to expand and fortify an agile supply chain which enhances reputation. Moreover, variety of products will increase the number of clients because product variety will expand the number of products the market will accept.

Agility enhances product variety, lower distribution and storage costs, and asset specificity stimulates commitment, both elements achieve customer satisfaction, being key elements to construct and maintain the market place reputation and key buyers in the network.

Horizontal collaboration also contributes to build different reputation levels within the market place, which at the same time affect or determine the willingness to develop relationships with a particular wholesaler.

Agility through collaboration and flexibility is a necessary resource in food chains, not only to maintain a positive reputation, but as a management tool in food supply chains and to be in accordance with the dynamic seafood market changes.

In spite of the achievements of this research there were also some limitations. The most important limitation is the common problem of studying real-life networks: the difficulties in obtaining real data. Most network partners were very concerned about retaining commercial confidentiality. Another important limitation relates to fieldwork and the inability to visit and interview all network partners.

Nevertheless, we also demonstrated that qualitative approaches provide important tools that can help to understand the mechanisms and processes influencing the operation and function of the social network, complement the results from experimental models by analyzing real cases and looks beyond the statistics provided by the network structure.
